# Adherence to oral anticancer hormonal therapy in breast cancer patients and its relationship with treatment satisfaction: an important insight from a developing country

**DOI:** 10.1186/s12905-023-02276-5

**Published:** 2023-03-20

**Authors:** Amer A. Koni, Bushra A. Suwan, Maisa A. Nazzal, Alaa Sleem, Aiman Daifallah, Majd Hamed allah, Razan Y. Odeh, Sa’ed H. Zyoud

**Affiliations:** 1 Division of Clinical Pharmacy, Department of Hematology and Oncology, An-Najah National University Hospital, Nablus, 44839 Palestine; 2 Department of Clinical and Community Pharmacy, College of Medicine and Health Sciences, An-Najah National University, Nablus, 44839 Palestine; 3 Department of Hematology and Oncology, An-Najah National University Hospital, Nablus, 44839 Palestine; 4 Poison Control and Drug Information Center (PCDIC), College of Medicine and Health Sciences, An-Najah National University, Nablus, 44839 Palestine; 5 Clinical Research Center, An-Najah National University Hospital, Nablus, 44839 Palestine

**Keywords:** Adherence, Treatment satisfaction, Oral hormonal therapy, Breast cancer, Hormone-positive, Oral anticancer

## Abstract

**Background:**

Hormone-positive breast cancer is the most common type and represents a burden in all countries. Treatment satisfaction might be a predictor for adherence, as higher satisfaction with medication encourages patients to adhere appropriately to the medication and, consequently, successfully achieve the treatment goals. The present study evaluated the adherence of women with hormone-positive breast cancer to oral hormonal drugs and correlated it with treatment satisfaction and other sociodemographic and clinical factors.

**Methods:**

A cross-sectional design was applied. This study included two cancer centers. Data were collected from patients through face-to-face interviews and medical record reviews. The Medication Adherence Scale was adapted to assess medication adherence, and the Treatment Satisfaction Questionnaire for Medication (TSQM) version 1.4 was adopted to measure treatment satisfaction.

**Results:**

The final analysis included 106 patients, with a mean age ± SD of 51.9 ± 1.2. Approximately 35% were hospitalized in the past year. Current hormonal therapy among cancer patients included letrozole (38.7%), tamoxifen (31.1%), exemestane (17%), and anastrozole (13.2%). The median adherence score was 5.0 [4.8–6.0], and 62.3% adhered fully to their oral hormonal drugs in the past week. The median scores of effectiveness, side effects, convenience, and global satisfaction were 66.67 [61.11.0–72.22], 75.00 [48.44–100.00], 66.67 [66.67–72.22], and 71.43 [57.14–78.57], respectively. A significantly lower adherence score was identified in patients living in camps (*p* = 0.020). Patients with comorbidities and those who continued on the same hormonal therapy had higher adherence scores, although they were not statistically significant. Multiple linear regression analysis showed that two domains of treatment satisfaction, side effects (*p* = 0.013) and global satisfaction (*p* = 0.018), were predictors of adherence to oral hormonal drugs.

**Conclusions:**

The current study revealed a significant association between treatment satisfaction and adherence to oral hormonal therapy. We recommend creating a specialized scale to measure adherence, considering the psychosocial factors that affect hormonal anticancer medication adherence.

## Background

In modern times, breast cancer is one of the most common health conditions faced by women worldwide [1]. It represents approximately 24.5% of all types of cancer in females and affects 1 in 8 women during their lifetime [1]. In Palestine, breast cancer is the most common cancer. In 2021, the number of new breast cancer cases in Palestine was 876 [2]. Based on statistics in the United States, Hormonal positive/human epidermal growth factor receptor 2 (HER2) negative breast cancer is the most common subtype and represents approximately 68% [3]. Oral hormonal anticancer drugs (i.e., tamoxifen and aromatase inhibitors) are prescribed for women with estrogen-positive and progesterone-positive breast cancer, with a highly satisfactory result after using these treatments [4]. It is often started as an adjuvant treatment following surgery/radiotherapy/chemotherapy or a combination of these therapies and given for 5 to 10 years [5]. It can also be given as a neo-adjuvant [5].

In a systematic review, the adherence rate to adjuvant hormonal therapy was approximately 66% [6]. Furthermore, it was found that more than half of breast cancer patients had nonadherent behavior to their treatment [7]. This percentage is close to that in Arabic nations, where Saudi Arabia reported a 69% adherence rate to antihormonal therapy [8]. Depression, older age, comorbidities, younger age, and side effects were associated with lower adherence. However, therapy with aromatase inhibitors, received chemotherapy, and prior medication use were associated with improved adherence [6]. In addition, it was found that adherence to hormone therapy increases disease-free survival [9]. Nevertheless, adherence to endocrine treatment decreased with years of therapy [10].

A previous study proved that satisfaction with oral anticancer drugs substantially affects adherence [11]. Breast cancer is a notable burden in all countries, and its incidence is high [12]. Suppose a cancer patient follows the treatment plan and adheres to the medications directed by his physician. In that case, it improves the survival rate and decreases the likelihood of recurrence [13]. Patient satisfaction with treatment is the key that encourages him to adhere to medications and successfully achieve short- and long-term results [11]. However, treatment satisfaction assessment helps healthcare professionals know the exact level of their patient's satisfaction with a specific drug and subsequently modify the treatment plan or find other solutions. This study will be the foundation for other projects that aim to evaluate adherence and treatment satisfaction in different cancer populations or with other therapies. There are limited reports on endocrine therapy adherence and treatment satisfaction in Palestine. Therefore, this study aims to determine the adherence rate and study factors associated with adherence.

## Methods

### Study design and sampling technique

We conducted the current multicenter cross-sectional study to assess breast cancer patients' adherence and satisfaction with oral hormonal medications using two main sets of data: medical records (both on paper and electronic) and women breast cancer patients’ interviews. This research was conducted using convenience sampling between November 2021 and January 2022. All patients who came to the hospital for treatment or follow-up care and met the inclusion criteria were asked to complete the questionnaire.

### Study setting

Our research was carried out in the oncology centers of the Al-Watani Hospital and the An-Najah National University Hospital in Nablus, Palestine. These hospitals are the largest and most important referral sites for cancer patients from all locations in Palestine.

### Sample size

According to medical records, the number of women with breast cancer visiting the two hospitals during the study period was approximately 175. Therefore, the recommended sample size was 121 patients using an online calculation, Raosoft, with a response of 50%, a 5% margin of error, and a 95% confidence interval.

### Exclusion and inclusion criteria

This study included women who had survived breast cancer over 18 years of age and had been prescribed and initiated oral hormonal drugs (neoadjuvant or adjuvant) at least four weeks prior to enrollment. Patients with comorbid delirium, dementia, bipolar, substance dependence disorders, untreated psychotic disorders, hospitalized patients, or those who were unable to participate or refused were excluded because of their inability to consent. We also excluded patients with missing findings in their medical records.

### Data collection instrument and procedure

Two clinical pharmacists collected the data through face-to-face interviews with patients. Before beginning the data analysis, regular checks were performed for data integrity, proper sequences of information, and an evaluation of missing or incomplete variables.

Questionnaires were completed by explaining the questions to the patients, filling in the information on papers using specific scales to assess cancer patients' adherence and satisfaction, and recording the patients’ sociodemographic information (Table 1). In addition, medical records were used to record information related to disease and treatment characteristics.

**Table 1:** Sociodemographic and clinical characteristics (*N* = 106)

Factor	Frequency (%)
**Age (years)**	
< 45	32 (30.2)
45–65	57 (53.8)
> 65	17 (16.0)
**Body mass index**	
< 18.5	2 (1.9)
18.5–24.9	25 (23.6)
25–29.9	37 (34.9)
≥ 30	42 (39.6)
**Residency**	
City	53 (50.0)
Village	45 (42.5)
Camp	8 (7.5)
**Smoking**	
No	89 (84.0)
Yes	17 (16.0)
**University qualification**	
No	71 (67.0)
Yes	35 (33.0)
**Work**	
Unemployed	89 (84.0)
Employed	17 (16.0)
**Material status**	
Single	23 (21.7)
Married	83 (78.3)
**Family history**	
No	46 (43.4)
Yes	60 (56.6)
**Comorbidities**	
No	51 (48.1)
Yes	55 (51.9)
**Chronic medication**	
No	33 (31.1)
Yes	73 (68.9)
**Number of clinic visits per year**	
< 12	29 (27.4)
≥ 12	77 (72.6)
**Hospitalized in the last year**	
No	69 (65.1)
Yes	37 (34.9)
**Pathology type**	
Lobular	8 (7.6)
Ductal	93 (87.7)
None	4 (3.8)
Both	1 (0.9)
**Breast surgery**	
No	20 (18.9)
Yes	86 (81.1)
**Radiotherapy**	
No	37 (34.9)
Yes	69 (65.1)
**Chemotherapy**	
No	21 (19.8)
Yes	85 (80.2)
**Biological therapy**	
No	86 (81.1)
Yes	20 (18.9)
**Targeted therapy**	
No	86 (81.1)
Yes	20 (18.9)
**Initial hormonal therapy**	
Tamoxifen	50 (47.2)
Exemestane	11 (10.4)
Letrozole	33 (31.1)
Anastrozole	12 (11.3)
**Current hormonal therapy**	
Tamoxifen	33 (31.1)
Exemestane	18 (17.0)
Letrozole	41 (38.7)
Anastrozole	14 (13.2)
**Hormonal drug switching**	
No	76 (71.7)
Yes	30 (28.3)
**Duration of starting current hormonal therapy**	
< 1 year	36 (34.0)
≥ 1 year	70 (66.0)
**HER2 status**	
Negative	82 (77.4)
Positive	24 (22.6)
**Disease recurrence**	
No	95 (89.6)
Yes	11 (10.4)
**Menopausal status**	
Premenopause	56 (52.8)
Postmenopause	50 (47.2)

### Medication adherence

The Medication Adherence Rating Scale (MARS) is used to assess adherence to medication [14]. It is a 10-item self-report instrument with yes/no responses to the questions asked, with a summation yielding a maximum of 10 points. MARS scores can range from 0 (low likelihood of adherence) to 10 (high likelihood of adherence). It also has three groups of items: "medication adherence behavior" (questions 1–4), "attitude toward taking medication" (questions 5–8) and "negative side effects and attitudes toward oral hormonal medication" (questions 9, 10). However, three theoretically irrelevant items (questions 5, 7, and 9) were removed due to poor item-total correlation. These excluded items are “I take my medication when I am sick”, “My thoughts are clearer on medication”, and “I feel weird, like a 'zombie', on medication”. Therefore, the maximum point became 7 (high likelihood of adherence).

### Treatment satisfaction

The Treatment Satisfaction Questionnaire for Medication (TSQM) version 1.4 assesses patients' perceptions of treatment [15–18]. It evaluates effectiveness (items 1–3), side effects (items 4–8), convenience (items 9–11), and global satisfaction (items 12–14). The TSQM is a validated scale ranging from 0 to 100, with a higher score denoting better satisfaction [19]. The TSQM scale uses 14 questions to evaluate patient satisfaction; questions 1 through 3 inquire about the patient's satisfaction with the drug's efficacy in preventing and treating his disease, as well as the drug's capacity to relieve the patient's symptoms and the length of time it takes to begin working. Questions 4–8 inquire about the drug's adverse effects, the degree to which the patient finds them bothersome, how they affect his bodily and emotional well-being, and how much of an impact they have on the patient's satisfaction with the medication. The ninth and tenth questions concern the ease or difficulty of using the medication and scheduling a time for it to be used, while the eleventh question concerns whether it is proper to take the medication as directed. The confidence of the patient that this medication is helpful to him, that its benefits outweigh its drawbacks, and the degree of his general satisfaction with the medication are evaluated in questions 12 through 14. The Arabic version of the TSQM 1.4 is a valid and reliable instrument for assessing the perceptions of patients about treatment [19]. It has been used in several publications in Palestine [16, 17, 20–25]. In addition, IQVIA™ has given An-Najah National University permission to utilize this questionnaire in their research.

### Pilot study

The pilot study sample consisted of 10 breast cancer patients chosen at the same criteria as the study population. The questionnaire was also completed in the same manner as it was for the study's population. Both scales, TSQM and MARS, were tested in the sample to evaluate the simplicity, understandability, and time to fill out all questions of the questionnaire. The Cronbach's alpha was 0.673 for the effectiveness domain of TSQM, 0.899 for side effects, 0.747 for convenience, and 0.878 for global satisfaction.

### Ethical approval

The *Institutional Review Boards (IRB) of An-Najah National University* and the Palestinian Health Authority approved every aspect of the study protocol, including the use of and access to the patients' data. Furthermore, before initiating data collection, we properly explained all parts of the questionnaire to patients and received their verbal consent.

### Statistical analysis

The Statistical Package for Social Sciences (IBM-SPSS) version 21 was used to enter and analyze the data. The results were explained using frequencies and percentages. The sociodemographic and clinical characteristics were described using descriptive and comparative statistics. We expressed the continuous variables using the median and interquartile ranges because the data were not normally distributed, as tested by the Kolmogorov–Smirnov test. Therefore, the Mann‒Whitney U and Kruskal‒Wallis tests were applied to examine the differences between variables. The Spearman test (TSQM and MARS scores) determined the association between treatment satisfaction and adherence. After that, all documented significant variables (sociodemographics and treatment satisfaction domains) in univariate analysis were entered in multiple linear regression analysis to determine the predictors for adherence. It was determined that there was a significant association with the outcome variables if the *p* value was less than 0.05.

## Results

### Sociodemographic and clinical characteristics

Table 1 describes the sociodemographic and clinical characteristics of the 106 women with breast cancer. Of all 121 recruited patients, 15 refused to participate due to lack of time, privacy, and psychological problems. Approximately 53% of the participants were aged between 45–65 years, 39.6% were obese, 67% had no university education, and 84% were unemployed. According to clinical characteristics, most patients had comorbidities and took other chronic drugs (51.9% and 68.9%, respectively). Furthermore, 81.1% of the patients underwent breast surgery, while 80.2% received chemotherapy. The current hormonal therapy among cancer patients is as follows: letrozole 38.7%, tamoxifen 31.1%, exemestane 17%, and anastrozole 13.2% (Table 1).

### Description of associations between patient characteristics and adherence score

Among 106 women with breast cancer, the median adherence score was 5.0 [4.8–6.0] (range: 1.0–7.0). Approximately 62.3% of the patients reported a high likelihood of adherence to oral hormonal drugs in the past week. Regarding the associations between patient characteristics and adherence score, a significantly lower adherence score was identified in patients living in camps (*p* = 0.020). Patients with comorbidities and those who continued on the same hormonal therapy had higher adherence scores, although they were not statistically significant. In this study, patients with comorbidities had a mean rank of 58.18, with a median of 5.0 [5.0–6.0], while patients without comorbidities had a mean rank of 48.45, with a median of 5.0 [4.0–6.0]. In terms of hormonal drug switching, the mean rank of patients who switched to another hormone therapy was 45.18, with a median of 5.0 [4.5–6.0], while the mean rank of patients who continued with the same hormonal prescription was 56.78, with a median of 5.0 [4.5–6.0] (Table 2).

**Table 2:** Associations between patient characteristics and adherence score

Factor	Frequency (%)	Median [Q1-Q3]	Mean rank	*P* value
**Age (years)**				0.277
< 45	32 (30.2)	5.0 [4.0–6.0]	46. 67	
45–65	57 (53.8)	5.0 [5.0–6.0]	56.81	
> 65	17 (16.0)	5.0 [5.0–6.0]	55.26	
**Body mass index**				0.949
< 18.5	2 (1.9)	5.0 [5.0–6.0]	64.5	
18.5–24.9	25 (23.6)	4.0 [5.0–6.0]	52.02	
25–29.9	37 (34.9)	5.0 [5.0–6.0]	53.73	
≥ 30	42 (39.6)	4.0 [5.0–6.0]	53.65	
**Residency**				**0.020**
City	53 (50.0)	5.0 [5.0–6.0]	54.88	
Village	45 (42.5)	5.0 [4.5–6.0]	56.77	
Camp	8 (7.5)	4.0 [3.25–4.75]	26.00	
**Smoking**				0.360
No	89 (84.0)	5.0 [5.0–6.0]	59.41	
Yes	17 (16.0)	5.0 [4.0–6.0]	52.37	
**University qualification**				0.619
No	71 (67.0)	5.0 [4.0–6.0]	52.51	
Yes	35 (33.0)	5.0 [5.0–6.0]	55.50	
**Work**				0.809
Unemployed	89 (84.0)	5.0 [4.5–6.0]	53.20	
Employed	17 (16.0)	5.0 [4.5–6.0]	55.06	
**Material status**				0.468
Single	23 (21.7)	5.0 [5.0–6.0]	57.39	
Married	83 (78.3)	5.0 [4.0–6.0]	52.42	
**Family history**				0.399
No	46 (43.4)	5.0 [4.0–6.0]	50.78	
Yes	60 (56.6)	5.0 [5.0–6.0]	55.58	
**Comorbidities**				0.085
No	51 (48.1)	5.0 [4.0–6.0]	48.45	
Yes	55 (51.9)	5.0 [5.0–6.0]	58.18	
**Chronic medication**				0.060
No	33 (31.1)	5.0 [4.0–6.0]	45.61	
Yes	73 (68.9)	5.0 [5.0–6.0]	57.07	
**Number of clinic visits per year**				0.264
< 12	29 (27.4)	5.0 [4.0–6.0]	48.36	
≥ 12	77 (72.6)	5.0 [5.0–6.0]	55.44	
**Hospitalized in the last year**				0.972
No	69 (65.1)	5.0 [4.0–6.0]	53.43	
Yes	37 (34.9)	5.0 [5.0–6.0]	53.64	
**Breast surgery**				0.986
No	20 (18.9)	5.0 [4.25–6.0]	53.60	
Yes	86 (81.1)	5.0 [4.75–6.0]	53.48	
**Radiotherapy**				0.894
No	37 (34.9)	5.0 [4.5–6.0]	52.99	
Yes	69 (65.1)	5.0 [4.5–6.0]	53.78	
**Chemotherapy**				0.246
No	21 (18.9)	6.0 [5.0–6.0]	60.10	
Yes	85 (80.2)	5.0 [4.0–6.0]	51.87	
**Biological therapy**				0.915
No	86 (81.1)	5.0 [4.0–6.0]	53.35	
Yes	20 (19.8)	5.0 [5.0–6.0]	54.13	
**Targeted therapy**				0.676
No	86 (81.1)	5.0 [5.0–6.0]	54.07	
Yes	20 (18.9)	5.0 [4.0–6.0]	51.05	
**Current hormonal therapy**				0.825
Tamoxifen	33 (31.1)	5.0 [4.0–6.0]	50.08	
Exemestane	18 (17.0)	5.0 [4.0–6.0]	54.86	
Letrozole	41 (38.7)	5.0 [5.0–6.0]	56.22	
Anastrozole	14 (13.2)	5.0 [4.0–6.0]	51.86	
**Hormonal drug switching**				0.064
No	76 (71.7)	5.0 [4.5–6.0]	56.78	
Yes	30 (28.3)	5.0 [4.5–6.0]	45.18	
**Duration of starting current hormonal therapy**				0.303
< 1 year	36 (34.0)	5.0 [4.0–6.0]	49.44	
≥ 1 year	70 (66.0)	5.0 [5.0–6.0]	55.59	
**HER2 status**				0.690
Negative	82 (77.4)	5.0 [4.75–6.0]	54.11	
Positive	24 (22.6)	5.0 [4.25–6.0]	51.42	
**Disease recurrence**				0.861
No	95 (89.6)	5.0 [5.0–6.0]	53.33	
Yes	11 (10.4)	5.0 [4.0–6.0]	54.95	
**Menopausal status**				0.478
Premenopause	56 (52.8)	5.0 [4.0–6.0]	51.61	
Postmenopause	50 (47.2)	5.0 [5.0–6.0]	55.62	

### Description of the association between treatment satisfaction and adherence

As shown in Table 3, there were significant correlations between MARS score and treatment satisfaction, including side effects (*p* = 0.024) and global satisfaction (*p* = 0.008). Women with a high adherence rate had higher satisfaction scores than women with a low adherence rate. Spearman’s rank order correlation coefficient between MARS adherence score and side effects and global satisfaction TSQM scores indicated significant positive correlations (*r* = 0.220 and 0.258, respectively).

**Table 3:** Spearman’s correlations between treatment satisfaction and adherence

		**Effectiveness**	**Side effects**	**Convenience**	**Global satisfaction**
**MARS Score**	**Correlation Coefficient**	0.128	0.220^*^	0.164	0.258^**^
	***P* value**	0.192	**0.024**	0.092	**0.008**

^**^The correlation is significant at the 0.01 level (2-tailed)

^*^The correlation is significant at the 0.05 level (2-tailed)

### Description of associations between patient characteristics and treatment satisfaction

As shown in Table 4, the TSQM score assesses perceived effectiveness, side effects, convenience, and global satisfaction. The median score of each domain was 66.67 [61.11.0–72.22], 75.00 [48.44–100.00], 66.67 [66.67–72.22], and 71.43 [57.14–78.57], respectively. Postmenopausal patients had significantly higher satisfaction towards side effects (*p* = 0.049). In addition, patients with comorbidities had a higher global satisfaction score (*p* = 0.010). Furthermore, the satisfaction score toward side effects was significantly lower in patients with experienced side effects (*p* = 0.001) and those hospitalized in the last year (*p* = 0.030). Moreover, letrozole therapy was significantly associated with higher satisfaction with perceived effectiveness (*p* = 0.002) and global satisfaction (*p* = 0.004).

**Table 4:** Associations between patient characteristics and treatment satisfaction

**Factor**	Effectiveness **Median [Q1-Q3]**	***P* value**	Side effects **Median [Q1-Q3]**	***P* value**	Convenience **Median [Q1-Q3]**	***P* value**	Global satisfaction **Median [Q1-Q3]**	***P* value**
**Age (years)**		0.301		0.185		0.230		0.140
< 45	66.67 [55.56–72.22]		71.88 [43.75–87.50]		66.67 [61.11–75.00]		71.43 [42.86–71.43]	
45–65	66.67 [61.11–77.78]		81.25 [43.75–100.00]		66.67 [66.67–72.22]		71.43 [64.29–78.57]	
> 65	66.67 [61.11–76.39]		90.63 [68.75–100.00]		66.67 [66.67–76.39]		71.43 [57.14–76.79]	
**Body mass index**								
< 18.5	66.67		56.25		75.00		85.72	
18.5–24.9	66.67 [61.11–75.00]		87.5 [56.25–100.00]		66.67 [63.89–69.45]		71.43 [67.86–75.00]	
25–29.9	66.67 [61.11–72.22]		81.25 [53.13–100.00]		66.67 [66.67–75.00]		71.43 [57.14–71.43]	
≥ 30	66.67 [61.11–75.00]		75.00 [37.50–100.00]		66.67 [66.67–72.22]		71.43 [53.57–78.57]	
**Residency**		0.415		0.766		0.896		0.559
City	66.67 [61.11–72.22]		84.38 [45.31–100.00]		66.67 [66.67–77.78]		71.43 [57.14–71.43]	
Village	66.67 [61.11–77.78]		75.00 [43.75–100.00]		66.67 [66.67–72.22]		71.43 [57.14–78.57]	
Camp	66.67 [61.11–70.83]		68.75 [64.06–98.44]		66.67 [66.67–70.83]		64.29 [51.79–71.43]	
**Smoking**		0.350		0.982		0.325		0.922
No	66.67 [61.11–72.22]		78.13 [50.00–100.00]		66.67 [66.67–72.22]		71.43 [57.14–76.79]	
Yes	66.67 [61.11–83.33]		75.00 [40.63–100.00]		66.67 [66.67–77.78]		71.43 [57.14–78.57]	
**University qualification**		0.646		0.225		0.385		0.699
No	66.67 [61.11–72.22]		81.25 [56.25–100.00]		66.67 [66.67–72.22]		71.43 [57.14–78.57]	
Yes	66.67 [55.56–73.61]		71.88 [35.94–100.00		66.67 [66.67–77.78]		71.43 [57.14–71.43]	
**Work**		0.626		0.414		0.751		0.497
Unemployed	66.67 [61.11–72.22]		75.00 [45.31–100.00]		66.67 [66.67–72.22]		71.43 [57.14–71.43]	
Employed	66.67 [55.56–83.33]		87.50 [46.88–100.00]		66.67 [61.11–77.78]		71.43 [60.72–78.57]	
**Material status**		0.601		0.136		0.941		0.058
Single	66.67 [61.11–77.78]		90.63 [67.19–100.00]		66.67 [61.11–73.61]		71.43 [69.65–78.57]	
Married	66.67 [61.11–72.22]		75.00 [43.75–100.00]		66.67 [66.67–72.22]		71.43 [57.14–71.43]	
**Family history**		0.197		0.096		0.324		0.451
No	66.67 [61.11–77.78]		75.00 [43.75–93.75]		66.67 [61.11–69.45]		71.43 [57.14–78.57]	
Yes	66.67 [61.11–72.22]		87.50 [50.00–100.00]		66.67 [66.67–72.22]		71.43 [57.14–71.43]	
**Comorbidities**		0.543		0.750		0.685		**0.010**
No	66.67 [61.11–72.22]		75.00 [43.75–100.00]		66.67 [66.67–77.78]		71.43 [57.14–71.43]	
Yes	66.67 [61.11–77.78]		78.13 [48.44–100.00]		66.67 [66.67–72.22]		71.43 [64.29–78.57]	
**Chronic medication**		0.911		0.862		0.799		0.618
No	66.67 [61.11–72.22]		75.00 [43.75–100.00]		66.67 [66.67–77.78]		71.43 [57.14–71.43]	
Yes	66.67 [61.11–77.78]		78.13 [48.44–100.00]		66.67 [66.67–72.22]		71.43 [64.29–78.57]	
**Side effects (currently)**		0.523		**0.001**		0.766		0.153
No	66.67 [61.11–76.39]		100.00 [57.81–100.00]		66.67 [66.67–72.22]		71.43 [57.14–71.43]	
Yes	66.67 [61.11–72.22]		68.75 [40.63–87.50]		66.67 [63.89–72.22]		71.43 [64.29–78.57]	
**Number of clinic visits per year**								
< 12	66.67 [61.11–76.39]		75.00 [57.81–98.44]		66.67 [66.67–77.78]		71.43 [64.29–78.57]	
= > 12	66.67 [61.11–72.22]		81.25 [43.75–100.00]		66.67 [66.67–72.22]		71.43 [57.14–75.00]	
**Hospitalized in the last year**		0.329		**0.030**		0.972		0.154
No	66.67 [61.11–72.22]		87.50 [50.00–100.00]		66.67 [66.67–72.22]		71.43 [57.14–71.43]	
Yes	66.67 [61.11–77.78]		65.63 [32.81–93.75]		66.67 [61.11–76.39]		71.43 [66.08–78.57]	
**Breast surgery**		0.596		0.249		0.239		0.782
No	66.67 [61.11–72.22]		90.63 [56.25–100.00]		66.67 [66.67–76.39]		71.43 [58.93–78.57]	
Yes	66.67 [61.11–77.78]		75.00 [43.75–100.00]		66.67 [66.67–72.22]		71.43 [57.14–75.00]	
**Radiotherapy**		0.746		0.577		0.679		0.249
No	66.67 [61.11–77.78]		75.00 [37.50–100.00]		66.67 [66.67–72.22]		71.43 [57.14–78.57]	
Yes	66.67 [61.11–72.22]		81.25 [56.25–100.00]		66.67 [66.67–72.22]		71.43 [57.14–71.43]	
**Chemotherapy**		0.262		0.636		0.183		0.607
No	66.67 [56.95–70.83]		78.13 [56.25–100.00]		66.67 [66.67–76.39]		71.43 [51.79–71.43]	
Yes	66.67 [61.11–75.00]		75.00 [43.75–100.00]		66.67 [66.67–72.22]		71.43 [57.14–78.57]	
**Biological therapy**		0.222		0.651		0.648		0.348
No	66.67 [61.11–77.78]		75.00 [50.00–100.00]		66.67 [66.67–76.39]		71.43 [57.14–78.57]	
Yes	66.67 [51.39–70.83]		71.88 [39.06–100.00]		66.67 [66.67–76.39]		71.43 [51.79–71.43]	
**Targeted therapy**		0.693		0.693		0.863		0.453
No	66.67 [61.11–72.22]		81.25 [50.00–100.00]		66.67 [66.67–72.22]		71.43 [57.14–71.43]	
Yes	66.67 [61.11–77.78]		65.63 [39.06–100.00]		66.67 [66.67–76.39]		71.43 [57.14–78.57]	
**Current hormonal therapy**		**0.002**		0.051		0.356		**0.004**
Tamoxifen	66.67 [61.11–72.22]		75.00 [43.75–87.50]		66.67 [61.11–69.45]		71.43 [42.86–71.43]	
Exemestane	63.89 [54.17–68.06]		59.38 [29.69–100.00]		66.67 [66.67–77.78]		71.43 [55.36–78.57]	
Letrozole	72.22 [66.67–77.78]		87.50 [56.25–100.00]		66.67 [66.67–72.22]		71.43 [71.43–78.57]	
Anastrozole	61.11 [50.00–66.67]		100.00 [82.81–100.00]		66.67 [66.67–73.61]		57.14 [50.00–71.43]	
**Hormonal drug switching**		0.966		0.288		0.244		0.867
No	66.67 [61.11–72.22]		81.25 [45.31–100.00]		66.67 [66.67–77.78]		71.43 [57.14–78.57]	
Yes	66.67 [61.11–75.00]		68.75 [46.88–100.00]		66.67 [66.67–66.67]		71.43 [60.72–71.43]	
**Duration of starting current hormonal therapy**		0.541		0.620		0.533		0.755
< 1 year	66.67 [61.11–72.22]		75.00 [43.75–100.00]		66.67 [66.67–72.22]		71.43 [57.14–78.57]	
≥ 1 year	66.67 [61.11–77.78]		78.13 [48.44–100.00]		66.67 [66.67–73.61]		71.43 [62.50–73.22]	
**HER2 status**								
Negative	66.67 [61.11–72.22]		75.00 [50.00–100.00]		66.67 [66.67–72.22]		71.43 [57.14–78.57]	
Positive	66.67 [61.11–76.39]		75.00 [43.75–100.00]		66.67 [66.67–72.22]		71.43 [51.79–76.79]	
**Disease recurrence**		0.460		0.416		0.799		0.957
No	66.67 [61.11–72.22]		75.00 [43.75–100.00]		66.67 [66.67–72.22]		71.43 [57.14–78.57]	
Yes	66.67 [61.11–77.78]		81.25 [62.50–100.00]		66.67 [61.11–83.33]		71.43 [64.29–71.43]	
**Menopausal status**		0.610		**0.049**		0.309		0.159
Premenopause	66.67 [61.11–72.22]		75.00 [39.06–100.00]		66.67 [66.67–72.22]		71.43 [57.14–71.43]	
Postmenopause	66.67 [61.11–77.78]		87.50 [62.50–100.00]		66.67 [66.67–72.22]		71.43 [57.14–78.57]	

### Multivariate analysis of adherence score

From the univariate analysis, residency, side effects, and global satisfaction were found to be statistically significant (*p* < 0.05). Multiple linear regression analysis revealed that the side effects domain (*p* = 0.013) and global satisfaction (*p* = 0.018) were predictors of oral hormonal drug adherence (Table 5).

**Table 5:** Multivariate linear regression analysis of the adherence score

Model		Unstandardized Coefficients		Unstandardized Coefficients	t	Sig	95.0% Confidence Interval for B		95.0% Confidence Interval for B
		**B**	**Std. Error**	**Beta**			**Lower Bound**	**Lower Bound**	**VIF**
**1**	**(Constant)**	3.800	0.554		6.863	0.000	2.702	4.899	
	**Residency**	-0.259	0.153	-0.154	-1.691	0.094	-0.562	0.045	1.006
	**Side effects**	0.008	0.003	0.234	2.524	**0.013**	0.002	0.015	1.033
	**Global satisfaction**	0.016	0.007	0.223	2.406	**0.018**	0.003	0.030	1.036

^a^Dependent variable: Adherence Score

## Discussion

The current study examined the degree of adherence of Palestinian women with breast cancer to their oral hormonal therapy and described its correlation with treatment satisfaction and other variables.

The sample of our study represents the age of the breast cancer population, in which approximately half of the breast cancer cases in Palestine fall within the 45–65 age group [26]. Oral hormonal therapy has improved patients' overall survival in breast cancer and long-term outcomes. An important element of treatment success is adherence to the medication. In the current study, 62.3% adhered fully in the past week, with a median adherence score of 5.0 [4.8–6.0]. In general, the adherence rate to oral hormonal drugs ranged from 45 to 95.7% [27]. In a systematic review, the mean rate of adherence at five years for the implementation phase was 66.2%, and the mean persistence was 66.8% [6].

Our results showed that women living in refugee camps were less adherent than those who resided in cities or villages. This could be due to low residential stability and social affluence. Patients with comorbidities had a higher adherence score, similar to a previous study [28]. This may be explained by the fact that patients with multiple comorbidities are aware of their diseases and the consequences of being nonadherent to medications. In addition, patients with other conditions may also use co-medication for these indications, which might stimulate them to take antihormonal therapy since they have a ‘cocktail’ to take and follow a medication scheme. Importantly, women who switched from their hormone drugs to another experienced less adherence to the new medication. Similar findings were reported in previous studies [29–31]. However, this finding should be further highlighted to identify the causes of switching and its effect on adherence. Our study found no significant differences in adherence scores between the hormonal drugs used. Similarly, a study did not show a significant association between the adherence of patients using tamoxifen and those receiving aromatase inhibitors [32]. However, our results contradict those of previous studies related to educational level, radiation therapy, age, and hospitalization, all of which were found to be significantly associated with adherence to hormonal therapy [33, 34].

Concerning treatment satisfaction, we found that Palestinian patients had different scores in the four domains of treatment satisfaction, with lower scores in effectiveness and convenience. Patients on oral hormone therapy may not objectively feel an improvement in their health. Furthermore, the long duration of this therapy (5–10 years) may impact treatment satisfaction. However, the treatment satisfaction domain score of side effects was significantly lower in patients with experienced side effects or hospitalization in the past year. It was evident that side effects substantially decreased the patient’s satisfaction with treatment.

In this study, treatment satisfaction (side effect and global satisfaction domains) was a predictor of adherence to oral hormonal drugs. This finding means that a high adherence score is associated with low experienced side effects and high global satisfaction rates. A previous study found that greater satisfaction with treatment led to more adherence to oral cancer drugs, including hormonal medications [11]. However, another study revealed no obvious correlation between adherence and patient satisfaction with medication information. The domain of side effects represented an essential impact on treatment satisfaction and adherence. Adverse effects from hormonal therapy were considered the main barrier to nonadherence [28, 34–36], and it negatively impacts the quality of life [32]. In our study, the highest beta coefficient was for the variable side effects. This suggests that side effects contributed the most to explaining differences in hormonal drug adherence.

Our result is close to other studies, which indicated a considerably high percentage of nonadherence [32, 33, 37]. Clinicians should pay great attention to this issue, as nonadherence is correlated with all-cause mortality in Asian women with breast cancer [38]. For example, physician‒patient and pharmacist-patient communications should be enhanced [39] or an app-based new technique, such as a smartphone intervention [40] or using bubble packaging [41], should be adopted.

## Strengths and limitations

This is the first study to correlate adherence and treatment satisfaction in patients with breast cancer treated with oral hormonal drugs and to analyze twenty-five sociodemographic and clinical factors. However, a cross-sectional design, a small sample size, the inclusion of only two centers, using self-report questionnaires, and convenient selections are considered limitations of the current study, affecting our findings' generalizability. Additionally, certain factors, such as receiving counseling from an oncologist/clinical pharmacist about medications, time since onset treatment, and stages of the disease, were not analyzed, as these variables may have a notable impact on adherence. Furthermore, the TSQM scale was not validated in the Palestinian population. Finally, MARS was developed for a psychiatric population and was not validated in a cancer population. Although the MARS was set for psychiatric patients [14], it had convergent validity, biologically measured adherence, good internal consistency, and test–retest reliability. It has also been used in a previous study among cancer patients receiving oral anticancer agents [42]. Importantly, the adherence scale used in the current study was adapted by removing three irrelevant items from the original MARS scale.

## Conclusions

The current study found that higher treatment satisfaction, especially with regard to side effects, was strongly associated with good adherence to oral hormonal therapy. Adjuvant hormone therapy seems to be an exceptional situation for medication adherence because the relationship between psychosocial factors and adherence to hormonal therapy in breast cancer differs from the relationship in other chronic conditions [43]. Therefore, we recommend creating a specialized scale to measure adherence, considering the psychosocial factors that affect hormonal anticancer medication adherence. In addition, pharmacists should counsel cancer patients about hormonal therapy, addressing the reasons for nonadherence and handling them. Finally, awareness of healthcare professionals regarding oral hormonal drug adherence is the cornerstone to openly discussing risks for nonadherence with cancer patients.

## Data Availability

Due to privacy, the data sets used and/or analyzed during the current study are available from the corresponding author upon reasonable request.

## Abbreviations

MARS: Medication Adherence Rating Scale
TSQM: Treatment Satisfaction Questionnaire for Medication
HER2: Human epidermal growth factor receptor 2
BMI: Body mass index (BMI)
IRB: Institutional Review Boards
SPSS: Statistical Package for Social Sciences
